# Estimating the Prevalence and Genetic Risk Mechanisms of ARFID in a Large Autism Cohort

**DOI:** 10.3389/fpsyt.2021.668297

**Published:** 2021-06-09

**Authors:** Tanner Koomar, Taylor R. Thomas, Natalie R. Pottschmidt, Michael Lutter, Jacob J. Michaelson

**Affiliations:** ^1^Department of Psychiatry, The University of Iowa, Iowa City, IA, United States; ^2^Department of Psychology, Pennsylvania State University, State College, PA, United States; ^3^Eating Recovery Center of San Antonio, San Antonio, TX, United States

**Keywords:** autism, eating disorders, genetics, ARFID, heritability

## Abstract

This study is the first genetically-informed investigation of avoidant/restrictive food intake disorder (ARFID), an eating disorder that profoundly impacts quality of life for those affected. ARFID is highly comorbid with autism, and we provide the first estimate of its prevalence in a large and phenotypically diverse autism cohort (a subsample of the SPARK study, *N* = 5,157 probands). This estimate, 21% (at a balanced accuracy 80%), is at the upper end of previous estimates from studies based on clinical samples, suggesting under-diagnosis and potentially lack of awareness among caretakers and clinicians. Although some studies suggest a decrease of disordered eating symptoms by age 6, our estimates indicate that up to 17% (at a balanced accuracy 87%) of parents of autistic children are also at heightened risk for ARFID, suggesting a lifelong risk for disordered eating. We were also able to provide the first estimates of narrow-sense heritability (h^2^) for ARFID risk, at 0.45. Genome-wide association revealed a single hit near *ZSWIM6*, a gene previously implicated in neurodevelopmental conditions. While, the current sample was not well-powered for GWAS, effect size and heritability estimates allowed us to project the sample sizes necessary to more robustly discover ARFID-linked loci via common variants. Further genetic analysis using polygenic risk scores (PRS) affirmed genetic links to autism as well as neuroticism and metabolic syndrome.

## 1. Introduction

Parents and caretakers of young children often report “picky eating” as a major concern. Food selectivity (eating from a small range of foods) and food neophobia (refusing to try new foods), are commonly seen in children under the age of 6 and are associated with socioeconomic factors (1) as well as individual factors like food preferences, appetite, and parental feeding strategies (2). Currently, there is no consensus among clinical and research settings on the definition of picky eating, and multiple terms for feeding challenges are used in the pediatric feeding literature (3). As a result, there are differences in assessment and estimates of prevalence, which can affect treatment recommendations. Prevalence studies show about half (46%) of the general population of young children struggle with typical picky eating, as reported in a 2015 Dutch study (4), while roughly one in ten children are considered to have extreme picky eating: 13% in the Netherlands (4), 14% in the United States (5).

Although picky eating is common in the general population of young children, increased prevalence rates are seen in children with developmental disorders–particularly autism spectrum disorders (autism) (6). Specifically, a meta-analysis of prospective studies found that children with autism are 5 times more likely to have an eating concern than their typically developing (TD) peers (7), although clinically relevant negative eating behavior rates do begin to decrease by age 6 (8). The most common presentations of eating concerns in children with autism include eating from a very narrow range of foods, or only eating foods with a specific presentation or sensory characteristic (pickiness), and avoiding eating new foods (food neophobia) (6). Bandini and colleagues have specifically defined food selectivity in this population as defined by food refusal, a limited food repertoire, and high-frequency single food intake (having a diet overwhelmed by one particular food) (9). Although some feeding concerns are shown to wane with age (10), adolescents and adults with autism still report significantly higher eating/feeding concerns than their TD peers (11, 12).

Traditionally, the clinical relevance of restrictive meal patterns in the context of eating disorders has focused on the fear of fatness/drive for thinness, which excluded many patients without such concerns from diagnosis and treatment. In 2013, Avoidant Restrictive Food Intake Disorder (ARFID) was first included in the DSM-V as a feeding disorder, characterized by a persistent pattern of food avoidance, which leaves individuals unable to meet their nutritional needs. ARFID is distinct from anorexia, as individuals with ARFID typically do not fear weight gain (13), implicitly making it a broader, more inclusive diagnosis. ARFID is reported to be particularly comorbid with psychiatric disorders, including autism, ADHD, and anxiety disorders (13, 14). In adults with ARFID, the resulting eating behaviors may cause just as much (if not more) distress and impairment as eating disorders like anorexia and bulimia (15). Of children/adolescents either presenting for eating disorder evaluation or currently in treatment for eating/feeding behavior problems, 5–22% (depending on the study) meet diagnostic criteria for ARFID (16). Although no population-based prevalence estimates based on clinical assessment are available, a self-report questionnaire of primary school children in Switzerland estimated general prevalence of ARFID at 3.5% (14).

ARFID and autism have high comorbidity, with (16) finding that 13% of pediatric ARFID patients in their clinic had autism, despite the fact that “*…patients with longstanding feeding issues and autism are not typically admitted to our program…”*. Opportunistic estimates like this are critical to establish the over-representation of autism among individuals with ARFID, but they do not help provide an estimate of how many individuals with autism might be at risk for ARFID. Furthermore, relatively little remains known about how risk for each disorder might inform the other. For example, the primary drivers of ARFID are often categorized as: lack of interest in eating (appetite), avoidance due to sensory characteristics of food (pickiness), and anxiety over adverse consequences from eating like choking or vomiting (fear). Research on food selectivity in autism has typically focused on sensory sensitivities, but restricted and repetitive behaviors (RRBs) have also been found to strongly correlate with this phenomenon as well (17). While, the overlap of sensory sensitivities between autism and ARFID provides an obvious avenue for this comorbidity, how RRBs might increase risk for ARFID (or if they may make it more difficult to detect) remains understudied. For example, a narrative study found that because many parents believe eating problems are endemic in autism, they acquiesce to perceived “pickiness” (18), potentially failing to raise concerns until the effects of malnutrition are apparent. A clearer sense of how many individuals with autism are at risk for ARFID—as well as the particular behavioral patterns which may be indicative of this risk—may help clinicians and caregivers identify children with autism who should be referred for treatment.

Treatment of ARFID is complex, with a broad range of treatment options and settings (outpatient to intensive treatment programs), population (from neurotypical to primarily autism), and practitioner (psychologists, occupational therapists, speech and language pathologists, registered dietitian nutritionists). This heterogeneity can result in inconclusive diagnoses or inconsistent care plans (19). Treatment approaches also vary widely in part because there are very few randomized controlled trials with most clinical treatment based upon case reports/series or retrospective chart review (20). Common treatment approaches include: Cognitive Behavioral Therapy, Family Based Therapy, Responsive Feeding Therapy, Applied Behavioral Analysis, and Sequential Oral Sensory therapy. The lack of empirical treatments—coupled with the broad range of available options—can make it difficult for individuals and families to identify the most appropriate treatment. Therefore, further research at the intersection of AFRID and autism is warranted to better understand the root causes of feeding symptoms and to better inform treatment.

These prior studies beg several questions, which we set out to address in this investigation. First, what is the prevalence of ARFID in an autism sample and in their parents? Second, what is the eating profile of high risk ARFID individuals, and what core autism traits are most associated with ARFID risk? Lastly, what role does common genetic variation play in ARFID risk? This study was uniquely poised to address these questions due to the availability of genetic data and detailed phenotypic data on eating habits and problems in a large cohort of both individuals with autism (*N* = 5,157) and typically-developing parents (*N* = 4,985). The primary phenotypes utilized in assessing ARFID risk were the Nine-Item Avoidant/Restrictive Food Intake Disorder (ARFID) screen (NIAS) (21), as well as extensive questions on inflexible eating behaviors and sensory sensitivities, and familial history of ARFID and other eating disorders. This data, combined with genetic data and surveys related to core autism symptoms, allowed us to identify individuals at high-risk for ARFID and profile the associations of ARFID in the broader context of general eating habits, core autism traits, and common genetic variation.

## 2. Results

### 2.1. Factor Analysis of NIAS

Zickgraf and Ellis (21) identified three latent factors within responses to the NIAS in a typically developing population: picky eating, low appetite and fear. Within the autism-enriched sample of SPARK, we identified the same three factors in both probands and parents. The loadings of these factors were very similar between probands and parents (see Table 1).

**Table 1:** Factor loadings on the NIAS.

	**Picky factor**		**Appetite factor**		**Fear factor**	
**Question**	**Proband**	**Parent**	**Proband**	**Parent**	**Proband**	**Parent**
I am a picky eater	**0.83**	**0.83**	0.17	0.18	0.09	0.10
I dislike most of the foods that other people eat	**0.81**	**0.85**	0.19	0.18	0.11	0.10
The list of foods that I like and will eat is shorter than the list of foods I won't eat	**0.83**	**0.74**	0.15	0.21	0.07	0.14
I am not very interested in eating; I seem to have a smaller appetite than other people	0.21	0.24	**0.81**	**0.80**	0.18	0.19
I have to push myself to eat regular meals throughout the day, or to eat a large enough amount of food at meals	0.21	0.22	**0.82**	**0.79**	0.18	0.21
Even when I am eating a food I really like, it is hard for me to eat a large enough volume at meals	0.13	0.16	**0.76**	**0.74**	0.27	0.30
I restrict myself to certain foods because I am afraid that other foods will cause GI discomfort, choking, or vomiting	0.12	0.12	0.12	0.13	**0.84**	**0.81**
I eat small portions because I am afraid of GI discomfort, choking, or vomiting	0.07	0.10	0.25	0.30	**0.85**	**0.80**
I avoid or put off eating because I am afraid of GI discomfort, choking, or vomiting	0.08	0.14	0.26	0.25	**0.84**	**0.83**

*Bold values indicate the primary loadings of each question as previously identified by Zickgraf and Ellis (21)*.

### 2.2. Identification of Individuals at High Risk of ARFID

Because ARFID is believed to be under-diagnosed, we sought to identify individuals at high-risk for ARFID using the three NIAS factors—as well as all additional survey questions—using a logistic regression model to predict the small number of individuals who indicated they or their dependent had an ARFID diagnosis (53 probands and 35 parents). The fitted values from the models including the NIAS and survey questions (referred to hereafter as “ARFID Score”) performed better than a naïve predictor using only age and sex, as well as the NIAS questions alone (Supplementary Figure 1), with a balanced accuracy of 0.87 for parents and 0.80 for probands. To classify undiagnosed individuals as “high-risk” for ARFID (ARFID Risk Group), we set a cutoff on the ARFID Score corresponding to the point on the ROC curve which was closest to a perfect predictor (0,1) (indicated by the dotted line in Supplementary Figure 1A). Under this heuristic, 17% of parents and 21% of probands were predicted as being at high-risk for ARFID.

### 2.3. Profile of Individuals at High Risk of ARFID

**Figure 1:**
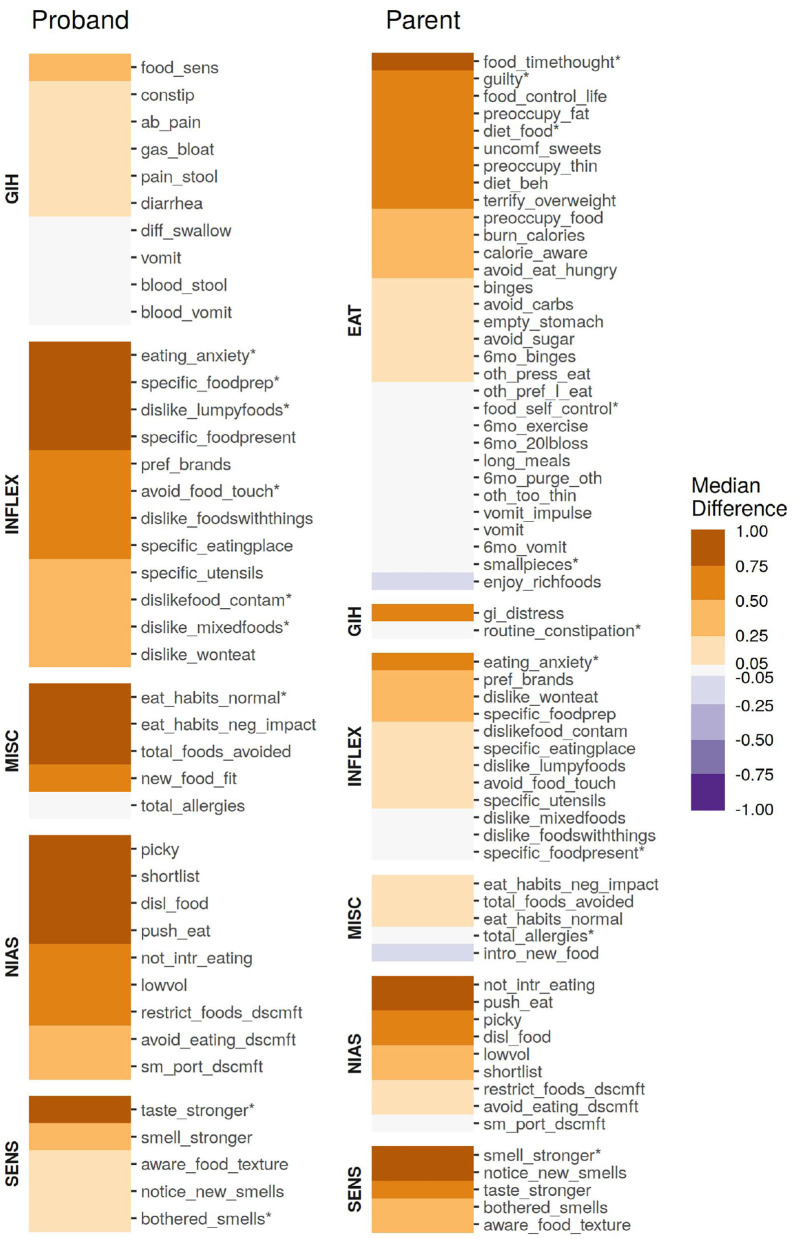
The association of individual age-corrected and scaled items from the parent or proband surveys, quantified as the median difference (location shift) from a Wilcox rank-sum test. All items were scaled to have a unit variance of 1 prior to quantification. Items marked with an asterisk (*) were included in the ARFID Score model.

The survey responses from parents and probands at high risk for ARFID exhibited distinct profiles, with some notable similarities, as seen in Figure 1, and described below. Unless otherwise stated, all reported associations had FDR *p* < 0.05.

#### 2.3.1. Nine-Item ARFID Screen (NIAS)

Although not directly included in either sub-cohort's ARFID risk model, the individual questions of the NIAS served as the foundation of both models. In both probands and parents, all nine items were significantly (*p* < 0.05, see Figure 1) associated with an endorsed ARFID diagnosis, though the questions underlying the “fear” factor was the least associated compared to the “picky” and “appetite” factors. In probands, three questions underlying the Picky Factor were the most enriched in high-risk individuals (Wilcox test location shift 95% CI range: 0.90–1.49 SD), while the questions underlying the Appetite Factor generally ranked higher in parents (Wilcox test location shift 95% CI range: 0.28–0.91 SD).

#### 2.3.2. Inflexible Eating Behaviors (INFLEX)

Anxiety over eating (“The idea of eating a food [parent/proband] does not like fills [her/him/me] with anxiety.”), most distinguished parents and probands identified as high risk from those at low risk for ARFID, (Wilcox test location shift 95% CI range: 0.70–0.77 SD for parents, 1.66–1.73 SD for probands). In fact, this item was included in both ARFID Score models (indicated by an asterisk in Figure 1). Inflexible behaviors were generally more enriched in high-risk probands compared to parents. This difference is particularly stark when it comes to presentation of food (“If a food that [parent/proband] usually likes is not presented in a certain way, [she/he/I] prefer(s) not to eat it.”).

#### 2.3.3. Sensory Sensitivities (SENS)

Sensory sensitives were found to be more broadly enriched in parents at high risk for ARFID than probands. In probands, sensitivity to taste was most pronounced in high-risk individuals (Wilcox test location shift 95% CI range: 0.64–0.88 SD). The opposite trend was found in parents, although the difference in enrichment was smaller than that observed in children.

#### 2.3.4. Gastrointestinal Issues (GIH)

Food sensitivities and problems with bowel movements were weakly enriched in high-risk probands (Wilcox test location shift 95% CI range: 0.09–0.49 SD). Meanwhile, issues with choking and vomiting were some of the weakest enrichment in high risk probands (Wilcox test location shift 95% CI range: 0.03–0.05 SD), which may be reflective of the slightly weaker enrichment of the items underlying the Fear Factor in this sub-cohort. Despite receiving a far less granular set of questions related to GI issues, general GI distress showed substantial enrichment in high-risk parents (Wilcox test location shift 95% CI range: 0.47–0.61 SD).

#### 2.3.5. Supplementary Questions (MISC)

Unsurprisingly, both high-risk parents and probands were more likely to have “abnormal” eating habits which negatively impacted day-to-day life—although only (“How much of a departure from the ‘norm' do you consider [proband/parent's] eating habits to be?”) was informative enough to be included in the proband ARFID risk model.

### 2.4. Association of NIAS Factors and ARFID Risk With Core Autism Symptoms

**Figure 2:**
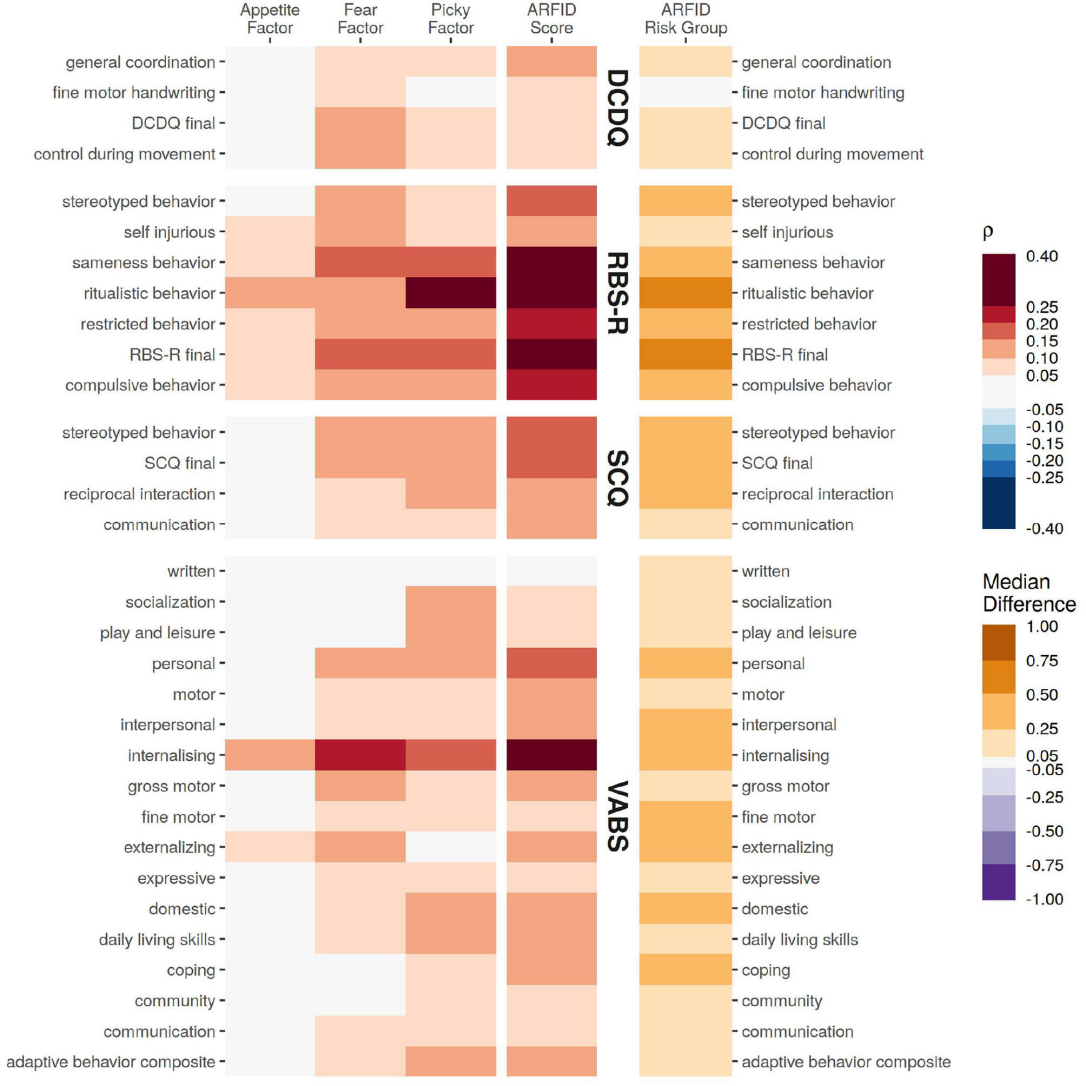
The association of various assessments from the probands with the NIAS factors and ARFID Score, quantified as Spearman correlation coefficient. For differences for those identified as high risk for ARFID (ARFID Risk Group), the association is quantified as the median difference (location shift) from a Wilcox rank-sum test.

The NIAS factors and ARFID score were found to be strongly associated with several measures of core autism symptomology (RBS-R, SCQ, and DCDQ) as well as adaptive behaviors (VABS). Unless otherwise stated, all associations reported below were FDR *p* < 0.05. Compared to the other NIAS factors, the Appetite Factor was found to have notably fewer—and weaker—associations (Figure 2).

Of the assessments covering core autism symptoms, restrictive and repetitive behaviors (RBS-R) had the strongest and broadest association, particularly with the ARFID Score (RBS-R total score ρ = 0.30, 95% CI: 0.28–0.32). Communication ability (SCQ) had more modest association with the ARFID Score (SCQ summary ρ = 0.18, 95% CI: 0.16–0.20), and had a stronger association with the Picky Factor (SCQ summary ρ = 0.15, 95% CI: 0.12–0.17), compared to the Fear Factor (SCQ summary ρ = 0.11, 95% CI: 0.09–0.13) or Appetite Factor (SCQ summary ρ = 0.02, 95% CI: −0.01 to 0.04, not significant). Developmental Coordination (DCDQ) had generally weaker correlations, the strongest of which were with the Fear Factor (DCDQ total ρ = 0.12, 95% CI: 0.09–0.15) and ARFID Score (DCDQ total ρ = 0.10, 95% CI: 0.07–0.13).

All of the adaptive skills assayed by the VABS—save for writing—were significantly associated with at least one NIAS factor or with ARFID risk. The strongest associations across all domains were with internalizing problems, (ARFID Score ρ = 0.30, 95% CI: 0.26–0.33, Fear Factor ρ = 0.21, 95% CI: 0.17–0.24, Picky Factor ρ = 0.17, 95% CI: 0.13–0.21, Appetite Factor ρ = 0.11, 95% CI: 0.07–0.15).

### 2.5. PRS

**Figure 3:**
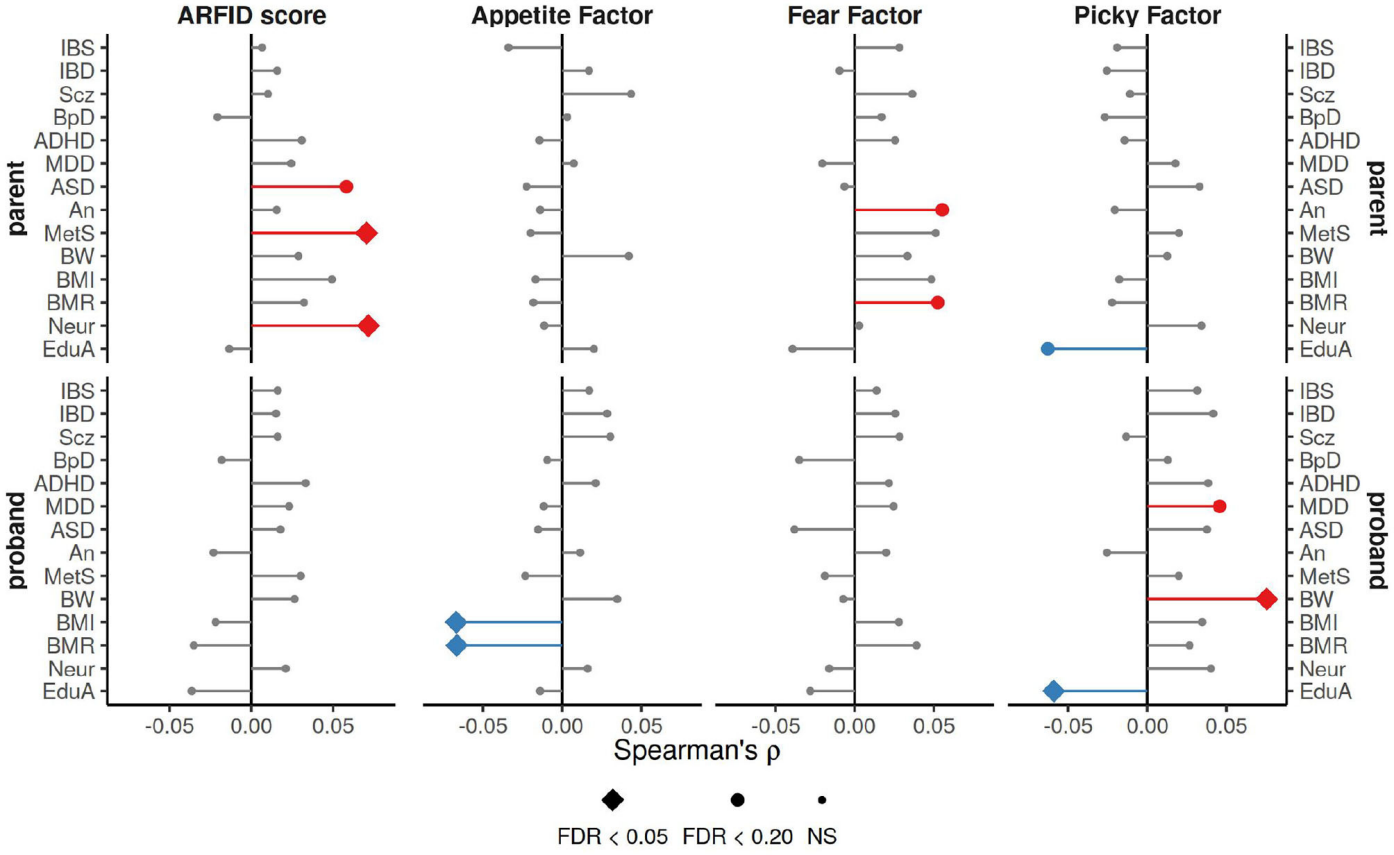
Spearman correlation coefficients for ARFID Score and NIAS factors with PRS for a variety of neuropsychiatric and morphological traits. The strongest association across three *p*-value thresholds (0.0005, 0.05, and 0.5), is shown here. FDR correction applied across all tests (*n* = 336). IBS, irritable bowel syndrome; IBD, inflammatory bowel disease; Scz, schizophrenia; BpD, bipolar disorder; ADHD, attention deficit hyperactivity disorder; MDD, major depression disorder; autism, autism; An, anorexia; MetS, metabolic syndrome; BW, birth weight; BMI, body mass index; BMR, basal metabolic rate; Neur, neuroticism; EduA, educational attainment.

The three NIAS factors and ARFID Score demonstrated genetic overlap with several neuropsychaitric and morphological traits, as measured by correlation with polygenic risk scores (PRS) for those traits (Figure 3). The ARFID Score of parents had significant positive association with PRS for metabolic syndrome (ρ = 0.070, FDR *p* = 0.03) and neuroticism (ρ = 0.072, FDR *p* = 0.03), and a nominal positive association with autism (ρ = 0.058, FDR *p* = 0.094). The Appetite Factor had significant negative correlations with BMI (ρ = −0.067, FDR *p* = 0.022) and basal metabolic rate (ρ = −0.066, FDR *p* = 0.022) in probands, while the Fear Factor was nominally positively correlated with basal metabolic rate (ρ = 0.052, FDR *p* = 0.194) and anorexia (ρ = 0.055, FDR *p* = 0.138) in parents. The Picky Factor was found to be negatively associated with PRS for educational attainment in both probands (ρ = −0.059, FDR *p* = 0.045) and parents (ρ = −0.062, FDR *p* = 0.057) and positively correlated with PRS for birth weight (ρ = 0.075, FDR *p* = 0.007) and nominally with major depression (ρ = 0.046, FDR *p* = 0.194) in probands alone.

### 2.6. Heritability

**Figure 4:**
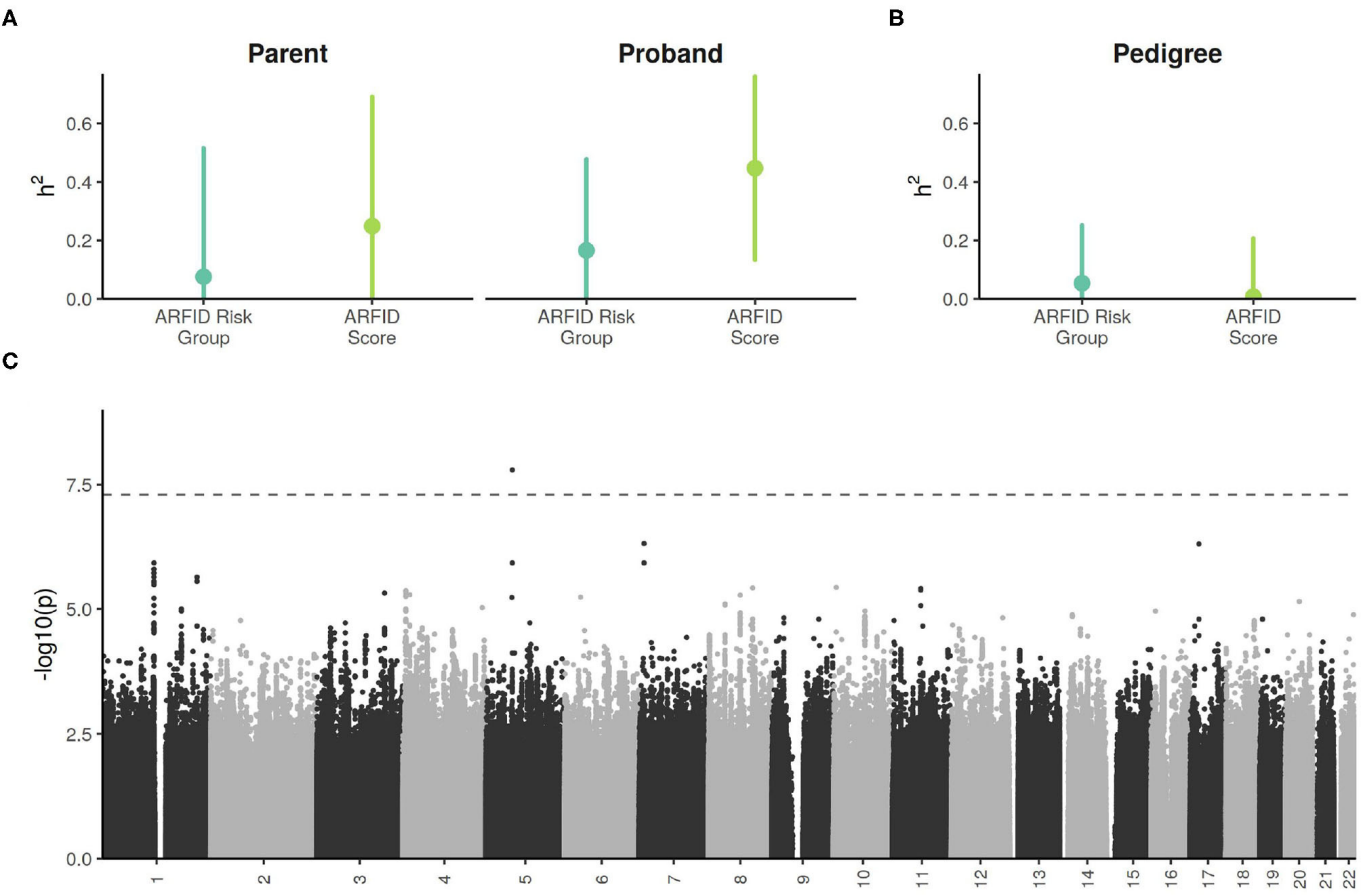
**(A)** SNP-based (i.e., narrow sense) heritability estimates of ARFID Score and ARFID Risk Group (high vs. low), with 95% confidence intervals. **(B)** Excess pedigree heritablility estimates (beyond what is explained by SNP h^2^) ARFID Score and ARFID risk group, with 95% confidence intervals. **(C)** GWAS Manhattan plot of the proband ARFID Score. The dashed line indicates the genome-wide significance threshold of 5*e*^−8^.

The size of each sub-cohort was too small detect narrow-sense (SNP-based) heritabilities of modest effect sizes, with a minimal detectable heritability (at 80% power) of 0.28 in the probands, and 0.40 in the parents (Supplementary Figure 2A). Despite this, the proband ARFID Score was estimated to have significant SNP h^2^ of 0.45 (95% CI: 0.13–0.76) (Figure 4A). The ARFID score for parents had a SNP h^2^ of 0.25 (95% CI: −0.19 to 0.69).

In the combined cohort, the h^2^ estimate for the ARFID score had little-to-no excess pedigree heritability (Figure 4B).

### 2.7. ARFID Score GWAS

One SNP on chromosome 5, rs13177031 (*p* = 1.6 × 10^−8^), reached genome wide significance (Figure 4C, Table 2). The gene nearest to this SNP is *ZWIM6* (13 kb downstream of SNP), which has been previously implicated in intellectual disability (22) and schizophrenia (23). A locus on chromosome 17 with the lead SNP rs73984121 (*p* = 5.0 × 10^−7^) is near *ULK2* (105 kb upstream of SNP) and *ALDH3A1* (17 kb downstream of SNP). *ULK2* has been shown to regulate axon growth in mice (24). A locus on chromosome 7 with the lead SNP rs78495856 (*p* = 4.9 × 10^−7^) is in an intron of the *THSD7A* gene. A previous genome-wide association study of BMI found an association with *THSD7A* (25).

**Table 2:** Top 5 lead SNPs from proband ARFID Score GWAS.

**rsID**	**Chr**	**Effect allele**	**Allele frequency**	**INFO score**	***P*-value**	**Beta**	**SE**	**Nearest genes**
rs13177031	5	A	0.35	0.83	1.6e-08	0.17	0.03	*ZSWIM6*
rs78495856	7	T	0.04	1.00	4.9e-07	0.32	0.06	*THSD7A*
rs73984121	17	T	0.03	0.95	5.0e-07	0.41	0.08	*ULK2, ALDH3A1*
rs1575620	1	C	0.50	0.96	1.2e-06	−0.13	0.03	*GDAP2, WDR3, SPAG17, TBX15, WARS2*
rs78624779	1	T	0.04	0.85	2.3e-06	−0.33	0.07	*GPATCH2, SPATA17*

The current sample is likely underpowered for effective gene discovery, which is supported by the discordance between the appreciable estimated SNP heritability and finding only one locus that exceeded the genome-wide significance threshold. Indeed, in a follow-up analysis that used the effect size and allele frequency of 162 independent lead SNPs from the GWAS, most would not be detectable as genome-wide significant without a cohort size of approximately 10,000 (Supplementary Figure 2B).

## 3. Discussion

The essential contributions of this work to our understanding of ARFID include the estimation of risk for ARFID in individuals with autism (21% of probands and up to 17% of their parents) and the demonstration of its heritability, estimated at 0.45 (95% CI: 0.13–0.76). We further implicated autism, metabolic syndrome, and neuroticism as genetically linked to ARFID via common variants, and identified *ZSWIM6*, a known neurodevelopment gene, as the first putative genetic association with ARFID. Our ARFID GWAS also suggested other genes implicated in BMI and axon growth, such as *ULK2* and *THSD7A*. Finally, although our GWAS was underpowered, our estimates of heritability allowed us to project the number of participants needed (*N* = 10,000) to achieve sufficient power for further ARFID gene discovery.

In line with previous work, three latent factors (appetite, fear, and pickiness) were found to underlie the NIAS in both the parent and proband sub-cohorts (21). These factors, along with other individual items from the parent and proband survey were useful in predicting ARFID diagnosis, resulting in an ARFID Score (Supplementary Figure 1), with a balanced accuracy of 0.85 in parents and 0.80 in probands. These estimates lead us to estimate that 17% of parents and nearly 21% of autism probands may be at high-risk for ARFID. These estimates are notably higher than one estimate of ARIFD rates in the general population of children at 3.5% (14), but are in line with an estimated ARFID prevalence of 22.5% among children undergoing treatment for eating disorders (16).

In contrast to previous studies of ARFID, which noted anxiety due to fears of choking, vomiting, or GI issues as the predominate drivers in a typically developing population, (16), our data indicate that RRBs and sensory sensitivities play a larger role, at least in autism. In probands, RRBs had stronger associations with ARFID risk than social skills, developmental coordination, and adaptive behaviors (Figure 2). RRBs also had the broadest association across all three of the NIAS factors, suggesting primacy in all domains of ARFID risk. Internalizing problems—a maladaptive subscale on the Vineland Adaptive Behavior Scales featuring questions about anxiety, worry, fear, and eating problems—had the next strongest association across all domains of that instrument. This raises the prospect that therapies targeted at RRBs or anxiety/fear may alleviate the factors underlying ARFID in some individuals with autism. Despite the strong inter-relationships of core autism symptoms (e.g., RRBs) with the fear and pickiness NIAS factors, the appetite NIAS factor lacked strong associations with any of the other adaptive, social, and coordination measures. This suggests a weaker interaction of low appetite with core autism symptoms, and consequently, less potential for inroads to treating ARFID via behavioral therapies when low appetite is the driving factor.

Given this apparent elevated risk for ARFID in autism, patterns of behavior associated with this risk—whether they involve eating or not—may be useful hallmarks for clinicians as they consider diagnoses. We found sensory sensitivities—particularly related to taste and food texture—to be over-represented in individuals at high risk for ARFID (Figure 1). This difference was less pronounced in autism probands than parents, perhaps due to the higher baseline amount of sensory sensitivity in individuals with autism. Thus, while these sensitivities clearly indicate ARFID risk, the presence of core autism symptoms may make them less salient.

Most neurodevelopmental conditions are heritable, with autism's SNP-based heritability estimated at 0.12 (95% CI: 0.10–0.14) (26) based on a cohort of 35,740 individuals. While, the continuous predictor of ARFID risk (ARFID Score) showed significant heritability in probands, the sample here was smaller by an order of magnitude (3,142 for probands and 2,205 for parents), leading to a less precise estimate: 0.45 (95% CI: 0.13–0.76) in probands and 0.25 (95% CI: −0.19 to 0.69) in parents. This indicates that common genetic variation likely plays a significant role in ARFID, especially in those with autism. We identified little excess pedigree heritability for the ARFID Score, suggesting that common genetic variation dominates environmental factors for ARFID risk. The GWAS and PRS analyses implicated both neurodevelopment and metabolism as significant factors involved in ARFID. The higher heritability in probands increased the power of GWAS, revealing one genome-wide significant SNP near the neurodevelopmental gene *ZSWIM6*. PRS analyses also revealed pleiotrophic associations of ARFID risk with neuroticism, autism, and metabolic syndrome in the parents. These associations may not have been detectable in probands because that sub-cohort was already enriched for autism and neuroticism risk. Further dissection of the ARFID score phenotype by the PRS associations of the NIAS factors showed the Appetite and Fear Factors to be more associated with metabolism, while the Picky Factor showed more associations with neurodevelopment (Figure 3).

### 3.1. Limitations

A primary limitation of nearly all studies in the SPARK cohort is the constraint of self- and parent-report surveys, rather than clinical assessment, to collect data. This means the length of a survey must be balanced with the burden on participants, often resulting in limitations of resolution and specificity. For example, because this study was focused specifically on a population-level study of ARFID, it was not well-situated to conclusively distinguish ARFID risk factors from those of other eating disorders. Regardless, this limitation is generally offset by the large sample sizes that can be recruited from within SPARK, and this work remains by far the largest study the authors are aware of to examine ARFID in the context of autism. Additionally, it should be noted that the NIAS (21), while designed as an adult self-report instrument, was adapted for use as a parent-report measure in this study due to a lack of empirically validated screening tools for ARFID in children at the time this research was conducted. The current study did demonstrate similar factor loadings of the three latent factors (picky eating, low appetite, and fear) in the NIAS across proband and parent subsamples. However, there may be error introduced in the probands' NIAS scores due to our reliance on parents' observation and interpretation of their children's eating habits.

A secondary limitation to reliance on self- and parent-report surveys in the context of eating problems is the effect of environment on the perception of eating habits. Parents in particular have extensive control over their environment, and may do so in order to minimize the adverse effects caused by sub-clinical eating problems, resulting in a bias of self-perception. There is also potential for ascertainment bias in reported ARFID diagnoses as a result of how some participants are recruited into SPARK. Although SPARK actively recruits across the entire United States, many participants are enrolled through partnerships with clinical sites (typically co-located with large research institutions). Due to data anonymization, we were unable to determine if self- and parent-reported ARFID diagnoses came from a small subset of clinical sites, where practitioners might be more aware of ARFID. However, such a scenario would simply reflect the overall under-diagnosis of ARFID, which was a major motivator for this study.

Particular features of the SPARK cohort also complicated comparisons between probands and parents due to representation of difference sexes in each. The Research Match survey for this study was sent to “primary” participants (independent adults with autism, or the parent who initiated enrollment into SPARK). In the data release use in this study, 88.6% of such “primary” parents are the biological mothers of probands. It is therefore possible that—although sex was accounted for in all tests performed here—the signal detected in parents is somewhat female-specific, just as the male-skewed proband subsample may lead to poor detection of patterns specific to female probands.

As a genome-wide genetic investigation, the current study had low power, though some important insight was obtained. We found that the current cohort was only suitable to detect substantial narrow-sense heritability (SNP h^2^ = 0.28–0.40; Supplementary Figure 2A). Similarly, the SNP-level associations found by GWAS were mostly below the detection threshold for the current cohort size (Supplementary Figure 2B). However, performing these analyses is an important first step in understanding the genetics of ARFID, as it allowed us to project that sample sizes of 10,000 and more would begin to yield returns, based on the effect sizes observed in our study.

### 3.2. Future Directions

This study showed the first evidence of significant SNP heritability of ARFID risk, and potential genetic connections to other traits, such as metabolic syndrome, autism, and neuroticism. Although the data yielded one genome-wide significant hit, this study is clearly underpowered, and future work will focus on expanding the sample size. Further, since previous studies have found evidence of rare variant burden in eating disorders (27–29), this is a logical next step in this cohort. Together, rare and common variant association studies may lead to a better understanding of the molecular pathways that underlie ARFID risk, potentially exposing new therapeutic opportunities. Finally, our estimators of ARFID do not explain all the variation in eating and gastrointestinal problems in our sample or in SPARK generally. As such, future work will concentrate on the identification of robust and recurrent patterns of eating and gastrointestinal phenotypes distinct from ARFID.

## 4. Materials and Methods

### 4.1. Cohort Description

Participants were recruited from the nation-wide SPARK study (30) via a research match. All respondents provided informed consent. This study was approved by the IRB of University of Iowa (IRB# 201801821) and SPARK is approved by the Western IRB (IRB# 20151664). In total, 5,686 independents responded to survey questionnaires for themselves and one proband. After merging the survey responses with available basic demographic and medical data, 5,157 probands and 4,985 adults without autism were used for analyses (Table 3). For analyses, these SPARK-collected data were combined with research match participants' survey responses on measures of eating behaviors and GI symptoms. A subset of 3,142 probands and 2,205 parents of European ancestry had SNP genotyping data available for analysis.

**Table 3:** Demographic summary of the cohort.

**Role**	**Proband**	**Parent**
Total *N*	5,157	4,985
Mean age (SD)	11.1 (5.87)	41.63 (8.24)
Male	81%	16%
Race: Asian	4%	–
Race: African American	7%	–
Race: Native American	3%	–
Race: Native Hawaiian	1%	–
Race: White	85%	–
Race: Other	5%	–
Cognitive impairment	17%	–
Mean BMI (SD)	–	29.84 (7.97)
Genotype *N*	3,142	2,205

*Variables with the mean reported have the standard deviation in parenthesis. Nine probands did not have data for cognitive impairment. Race was self-reported endorsement with none and/or multiple selections allowed*.

### 4.2. Phenotypic Measures

In addition to the measures assessed as part of this study (see below), all families also had available demographic, medical, and core autism behavioral data collected through SPARK. Except where noted, each primary respondent was a parent who answered the measures in our study for both self and their dependent (proband). Independent adults with autism were given the same survey as parents and answered the measures for themselves, but were not analyzed as part of this study.

#### 4.2.1. Nine-Item Avoidant/Restrictive Food Intake Disorder (ARFID) Screen (NIAS)

At the time this study was conducted, there was no validated measure for assessing symptoms of ARFID in children, so the NIAS (21) was adapted in the surveys to collect responses for parents as well as probands. The 9 items in this measure assess three categories of eating disturbance that can lead to ARFID symptoms, as described in the DSM-5: avoidance of many foods based on sensory properties, low appetite or limited interest in eating, and fear of negative consequences (i.e., choking, vomiting) from eating. The scale demonstrated high internal consistency (alpha ranging from 0.79 in an undergraduate sample to 0.90 in a sample with familial eating problem behaviors). One item in this measure simply asks respondents to self-identify picky eating behavior, which has been shown to distinguish picky from non-picky eaters in and of itself (31–34).

#### 4.2.2. Inflexible Eating Behavior

An Inflexibility Index was created by Zickgraf et al. (15) to measure the rigid eating behavior associated with picky eating severity, with items such as: “the thought of eating a food I do not like fills me with anxiety” and “I avoid letting different foods touch on my plate, even when they are both foods that I like.” An unrotated principal components analysis (PCA) indicated that all items loaded on a single factor, with all loadings >0.50, and the internal reliability for all 12 items was excellent (alpha = 0.92).

#### 4.2.3. Sensory Sensitivities

Additionally, Zickgraf et al. (15) developed 11 sensory sensitivity-related questions based upon questionnaires such as the SensOR Assessment (35) and the Glasgow Sensory Questionnaire (36). In an attempt to reduce the question load on participants of this study, we selected questions only related to food sensitivities (e.g., “my sense of taste/smell is stronger/more sensitive than other people's”; “I am very aware of the texture of food in my mouth”). These questions are designed to assess the sensory component of eating that might be affecting eating behavior in the study cohort.

#### 4.2.4. Eating Attitudes Test (EAT-26)

This shortened (26-item) version of the Eating Attitudes Test (originally 40 items) was developed in 1982 by Garner and colleagues, and has been used popularly as a screening measure for disordered eating behaviors since. Internal consistency of the EAT-26 is high (alpha = 0.90 in a cohort with anorexia nervosa and 0.83 for a group of female controls), and higher scores (>20) are indicative of potentially disordered eating (37). This measure was only included in the survey sets for parents.

#### 4.2.5. Gastrointestinal History (GIH)

The GIH assesses the frequency of 10 current gastrointestinal symptoms (within past 3 months). Assessing GI symptoms in individuals with autism can be very challenging due to potential lack of communicative abilities, and thus assessments so far have been varied, and none have yet been validated, according to a comprehensive literature review (38). However, of the 5 GI measures designed with an autism population in mind, Holingue and colleagues describe the CHARGE GIH as one of the simplest to take, while assessing a comprehensive range of GI symptoms and their frequencies. The GIH was only included in probands' survey sets; parents were simply asked to report the frequency with which they experience constipation or other gastrointestinal upset.

#### 4.2.6. Miscellaneous

Several standalone items were included in survey sets in order to assess some potentially important factors in eating behavior patterns: food allergies, foods avoided by preference, family history of eating disorders, and the subjective negative impact of eating behavior on daily life. The family history of eating disorders included the responding parent and proband (i.e., “self” history of eating disorders). ARFID diagnosis was based on this report by the responding parent.

#### 4.2.7. Measures of Broad Autism Symptoms

These data were released as part of SPARK Data Collection 5 in December 2020. For a breakdown of the summary and sub-scores from individual instruments, including sample number, see Supplementary Table 4. All data from SPARK, including autism diagnosis, are parent/caregiver or self-reported through online surveys.

##### 4.2.7.1. Social-Communication Questionnaire (SCQ)

The SCQ (39) was administered to probands and control siblings (when available), between the ages of 2 and 18 years. The final score was nullified when a surplus of missing items affected validity.

##### 4.2.7.2. Developmental Coordination Disorder Questionnaire (DCDQ)

The DCDQ (40) was administered for probands between the ages of 5 and 15 years. Scores were reversed so that higher scores indicated worse coordination. The DCDQ was not completed if parents/caregivers indicated the proband was unable to use his/her hands or to ambulate. The final score was nullified if one or more items were incomplete across an entire measure (in concordance with publisher recommendation).

##### 4.2.7.3. Repetitive Behavior Scale-Revised (RBS-R)

The RBS-R (41) was administered to dependent probands age 3 and older. Subscale scores were nullified if more than two component items were missing. The final score was nullified if more than 5 items total were missing.

##### 4.2.7.4. Vineland Adaptive Behavior Scales (VABS)

The Vineland 3rd Edition Parent-Caregiver Comprehensive rating form (42) was administered to parents or caregivers of probands using Pearson Q Global. Scores for all non-maladaptive scales were revered so that higher scores indicated more problems.

### 4.3. Phenotype Data Cleaning

Ordinal survey items were imputed to the median. 0.6% ordinal data was missing and imputed in the proband data, and 0.3% was missing in the parents. Ordinal survey items were corrected for non-linear effects of age via local regression. First, the empirical cumulative distributions of individual items were mapped to a standard normal distribution via ecdf() and qnorm() functions in R. Age was then residualized from responses using R's loess() function with span = 0.5.

### 4.4. Identification of Individuals at High Risk for ARFID

We performed a two-step process to create a quantitative score estimating every individual in the cohort's risk for ARFID and used this score to classify individuals as high or low risk. Both of these steps were carried out in parallel for parents and probands. The demographics, mean NIAS factor values, and median model items stratified by low risk and high risk ARFID groups are shown in Supplementary Table 2 for probands and Supplementary Table 3 for parents.

#### 4.4.1. Factor Analysis

Previous studies have reported three clear factors underlying the nine questions of the NIAS (21). Factors were calculated on the individual NIAS items using the factanal() function in R. Factor scores using Thompson's method were calculated for each individual. These factor scores were then scaled within each age decile (10 separate age bins for parents and probands), and these age-corrected factor scores were used as the phenotypes for all association analyses.

#### 4.4.2. Logistic Regression

To leverage the additional granularity of the survey questions described above, we fit logistic regression models predicting ARFID diagnosis with glm() in R, serially adding terms to the model to reduce the Akaike information criterion (AIC). The three NIAS factors were used as a baseline model and each additional survey item was added to the model, with the single item which reduced the AIC the most carried forward (Supplementary Table 1). This process repeated until none of the remaining survey questions reduced the models' AIC.

### 4.5. Common Genetic Variation

The SNPs used in this study were based on the combined SPARK 2019 Version 3 release and the SPARK 2020 Version 4 release.

#### 4.5.1. SNP Processing and Imputation

SNPs were merged using PLINK (43), then lifted from hg38 over to hg19. The SNP QC process was based on the recommendations by (44) using PLINK (43) and R (45). First, 25,840 SNPs and 86 individuals were removed due to global missing rate greater than 20%. Second, the more stringent threshold of 5% global missing rate was used again which removed an additional 30,845 SNPs and 711 individuals. Third, 102,530 SNPs were removed because the minor allele frequency was less than 1%. Fourth, 47,825 were removed due to the HWE *p*-value less than 1 × 10^−10^. Fifth, 1,180 individuals were removed because of missing rate greater than 5% on any autosome. Sixth, 1,115 individuals were removed due to their heterozygosity rate not within 3 standard deviations of the cohort mean heterozygosity. After this QC, the remaining SPARK cohort was merged and clustered with the 1,000 Genomes Phase 3.

Clustering was based on the first 10 components from multi-dimensional scaling of the combined kinship matrix of the cohort and 1,000 Genomes. This combined cohort was clustered into 5 groups, representing the 5 distinct super-populations. Three thousand nine hundred and sixty-three individuals were removed due to being more than 3 standard deviations away from any of the 5 mean multi-dimensional scaling components. In total, 36,154 individuals and 409,281 SNPs remained. The top 10 principal components of each of the 5 clusters of the SPARK cohort were calculated separately to be used as covariates in heritability, polygenic risk score, and genome-wide association analyses. These remaining individuals and SNPs were imputed to the 1,000 Genomes Phase 3 reference of their respective cluster using the Genipe pipeline (46). Genipe performed LD calculation and pruning with PLINK (43), genotype phasing with SHAPEIT (47), and genotype imputation by IMPUTE2 (48) using default parameters.

#### 4.5.2. Polygenic Risk Scores

Polygenic risk scores (PRS) were calculated using PRSice (49) from the following base GWASes: metabolic syndrome (UK Biobank 2019) (50), body mass index (UK Biobank 2019) (51), birth weight (UK Biobank 2019) (51), basal metabolic rate (UK Biobank 2019) (51), neuroticism (SSGAC 2016) (52), educational attainment (SSGAC 2018) (53), schizophrenia (PGC 2018) (54), major depression disorder (PGC 2018) (55), bipolar disorder (PGC 2018) (54), autism spectrum disorder (PGC 2019) (26), anorexia (PGC 2017) (56), attention deficit hyperactivity disorder (PGC 2019) (57), inflammatory bowel disease (58, 58), and irritable bowel syndrome (59). PRSes were calculated at three *p*-value thresholds: 0.0005, 0.05, and 0.5. To control for the possible effects of population stratification, the first 10 common genetic principal components were regressed out of each PRS using the lm() function in R. To control for any systematic differences in phenotype between sexes, all phenotypes were scaled separately by sex before association testing. The association of the NIAS factors and ARFID Score with each PRS was quantified as non-parametric (Spearman's) correlation using the cor.test() function in R. False discovery rate correction was applied to the *p*-values of these test across all PRS thresholds and scores/factors using the p.adjust() function in R.

#### 4.5.3. SNP Heritability

GCTA (60) was used to calculate SNP-based heritability, and the GCTA power calculator (61) was used to estimate the power of each sub-cohort (Supplementary Figure 2A). The genetic relationship matrix (GRM) was created for *N* = 29,443 individuals in the European population cluster. SNPs were pruned for a final set of 192,500 autosomal SNPs to generate the GRM. This resulted in *N* = 3,142 probands and *N* = 2,205 parents being used for GCTA. SNP-based heritabilities were calculated separately for parents and probands using the GREML method (62). The ARFID Score was scaled separately for males and females, and the first 10 scaled genetic principal components were used as covariates. Pedigree-based heritabilities on the combined phenotypes were calculated with both the original GRM and an additional GRM with a relatedness cutoff of 0.05 using the GREML method (63).

#### 4.5.4. Genome-Wide Association Study

The GWAS on the proband ARFID Score was performed on the directly observed and imputed SNPs using BOLT (64), a mixed-model analysis which accounts for population stratification and relatedness. The ARFID Score was scaled separately for males and females. The first 10 scaled genetic principal components were used as covariates. After the imputation quality control filtering performed by BOLT, 3,142 individuals were used for the GWAS. The summary statistics were filtered on 1% minor allele frequency and INFO imputation score of 0.8 or greater. 8,275,942 SNPs remained after filtering. Lead SNPs were identified using the default clumping parameters of plink (43). Estimates of the minor allele frequency and effect size detection threshold (at 80% power) in the proband sub-cohort was calculated using the genpwr package (65), assuming an additive genetic model (Supplementary Figure 2B).

### 4.6. Visualization and Data Processing

All visualizations were generated in R (45) with the ggplot2 (66) and patchwork (67) packages. Unless otherwise noted, data processing was performed in R using the tidyverse family of packages (68). When reported, 95% confidence intervals for Spearman correlations were based on 1,000 bootstrap permutations.

## Data Availability Statement

The datasets presented in this study can be found in online repositories. The names of the repository/repositories and accession number(s) can be found below: https://base.sfari.org, SFARI SPARK Research Match, RM0018. The code can be found below: https://research-git.uiowa.edu/michaelson-lab-public/spark-arfid-2021.

## Ethics Statement

The studies involving human participants were reviewed and approved by University of Iowa IRB #201801821. Written informed consent to participate in this study was provided by the participants' legal guardian/next of kin.

## Author Contributions

TT and TK performed all analyses and contributed equally to this publication. NP and JM conceived the study design and received all regulatory approvals. ML and NP performed background research. All authors drafted and provided revision of the manuscript for intellectual content.

## Conflict of Interest

The authors declare that the research was conducted in the absence of any commercial or financial relationships that could be construed as a potential conflict of interest.
