# Supplementary material for: Estimating the Prevalence and Genetic Risk Mechanisms of ARFID in a Large Autism Cohort
Source: Front Psychiatry. 2021 Jun 9;12:668297. doi: 10.3389/fpsyt.2021.668297 (PMC8221394; doi:10.3389/fpsyt.2021.668297)
Supplement: Supplementary file 1 [file Data_Sheet_1.PDF]

Supplementary Material

1 SUPPLEMENTARY TABLES AND FIGURES

1.1 Figures

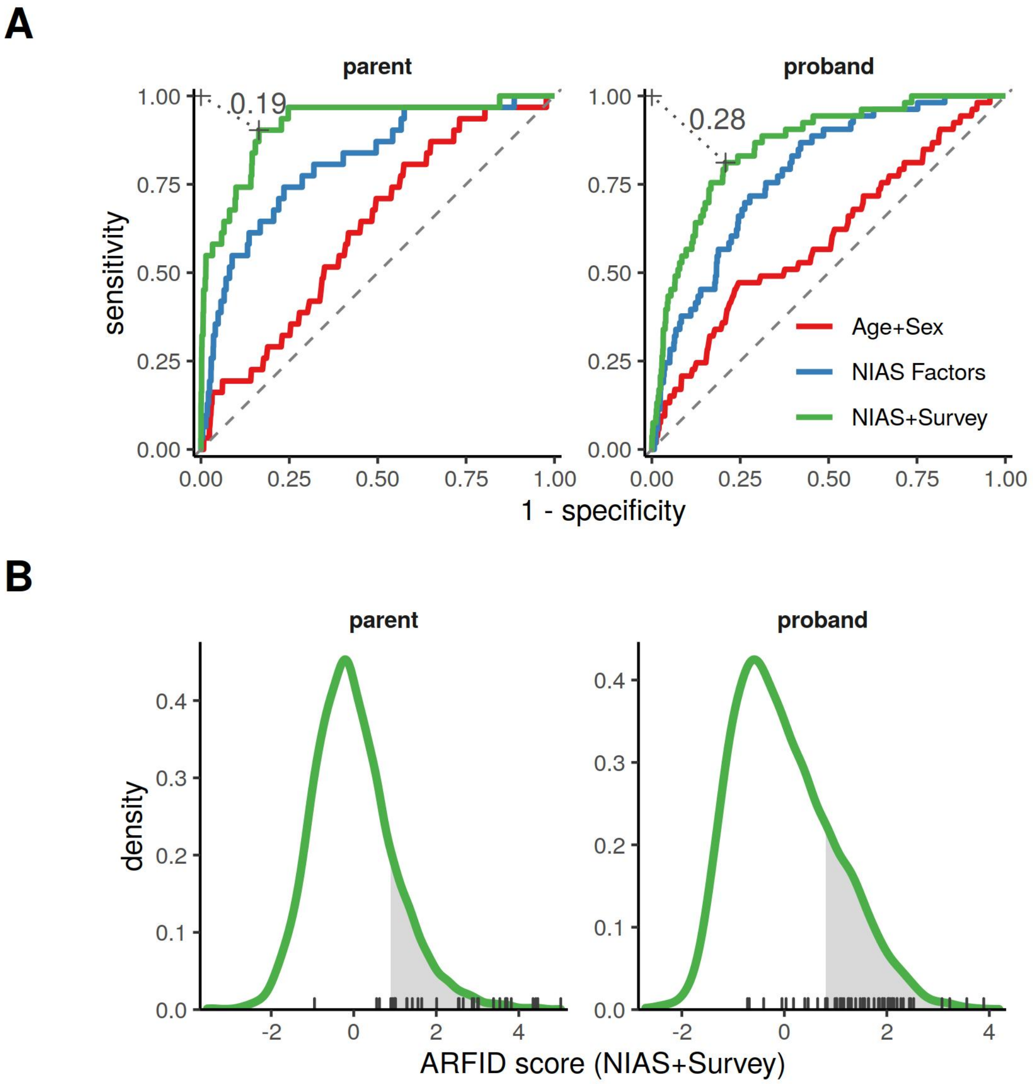

Figure S1. **A** ROC curves for the fitted values of logistic regression models predicting ARFID diagnosis within each sub-cohort. The point on each the NIAS+Survey line closest to (0,1) — a perfect predictor — is indicated by the dotted line flanked by cross-hairs. This is the threshold used to classify undiagnosed individuals as high or low risk for ARFID. **B** Distribution of ARFID scores for parents and probands. The shaded region under the curve indicates the threshold used to categorize individuals as high risk for ARFID (0.899 in parents and 0.803 in probands), and dark grey lines at bottom indicate individuals with a reported ARFID diagnosis.

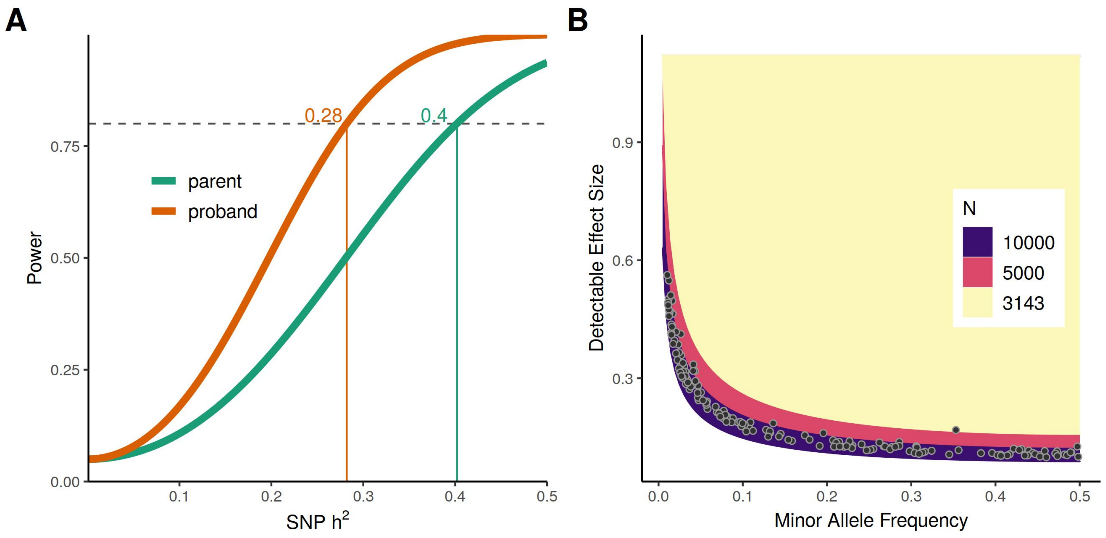

Figure S2. **A** Power curves to detect SNP-based heritability in the parent and proband sub-cohorts. Dashed line is at power = 0.8, vertical lines indicate heritability detection threshold for each sub-cohort. **B** Estimated minor allele frequency and effect size threshold to detect genome-wide significant SNPs in the proband sub-cohort with power = 0.8 at three different cohort sizes: 3,143 (current cohort size); 5,000; and 10,000. Points indicate independent lead SNPs from the GWAS of the ARFID risk score, mainly falling between the detection threshold of N=5,000 and N=10,000.

1.2 Tables

Source	Term	AIC	Z
**proband**			
NIAS	appetite factor	546.2	3.26
NIAS	fear factor	546.2	1.51
NIAS	picky factor	546.2	0.04
MISC	eat habits normal	535.9	2.81
INFLEX	eating anxiety	528.6	2.44
INFLEX	dislikefood contam	526.3	-2.75
INFLEX	specific foodprep	521.8	2.20
INFLEX	dislike mixedfoods	519.7	-2.65
INFLEX	avoid food touch	517.2	2.14
SENS	bothered smells	515.5	-2.25
INFLEX	dislike lumpyfoods	514.9	1.60
SENS	taste stronger	514.6	1.52
**parent**			
NIAS	appetite factor	343.7	3.78
NIAS	picky factor	343.7	1.82
NIAS	fear factor	343.7	0.41
EAT	food timethought	314.6	2.69
EAT	food self control	308.3	3.06
SENS	smell stronger	302.9	2.62
EAT	smallpieces	297.5	-3.25
EAT	diet food	289.0	2.54
EAT	guilty	286.5	2.15
INFLEX	eating anxiety	284.8	2.23
INFLEX	specific foodpresent	282.6	-2.06
GIH	routine constipation	281.6	1.71
MISC	total allergies	281.3	1.56

Table S1. Individual items included in ARFID scores for probands and parents, along with model AIC from recursive logistic regression when the specified term was added to the model. Z indicates the z-statistic from the final model fit which included all indicated terms.

	Low Risk	High Risk
Total (N)	4,040	1,117
Mean Age (SD)	11.19 (5.96)	10.79 (5.53)
Male	81%	81%
Race: Asian	5%	3%
Race: African American	7%	7%
Race: Native American	3%	4%
Race: Native Hawaiian	1%	1%
Race: White	85%	86%
Race: Other	5%	4%
Cognitive Impairment	17%	16%
Mean NIAS Appetite Factor (SD)*	-0.19 (0.79)	0.7 (1.31)
Mean NIAS Fear Factor (SD)*	-0.11 (0.87)	0.38 (1.29)
Mean NIAS Picky Factor (SD)*	-0.19 (0.95)	0.7 (0.85)
Median MISC eating habits normal (MAD)*	1 (0)	2 (0)
Median INFLEX eating anxiety (MAD)*	3 (1.48)	5 (0)
Median INFLEX dislike food contam (MAD)*	2 (1.48)	4 (1.48)
Median INFLEX specific food prep (MAD)*	3 (1.48)	5 (0)
Median INFLEX dislike mixed foods (MAD)*	3 (2.97)	5 (0)
Median INFLEX avoid foods touch (MAD)*	2 (2.97)	4 (1.48)
Median SENS bothered smells (MAD)*	2 (1.48)	3 (2.97)
Median INFLEX dislike lumpy foods (MAD)*	3 (2.97)	5 (0)
Median SENS taste stronger (MAD)*	3 (1.48)	5 (0)

Table S2. Proband demographic summary and ARFID model variables stratified by ARFID risk group. An asterisk * indicates the variable was included in the model. For the individual items included in the model, the raw variables are being reported (unimputed and not corrected for age/sex). Variables with the mean reported have the standard deviation in parenthesis. Variables with the median reported have the median absolute deviation in parenthesis. Nine probands did not have data for cognitive impairment. Race was self-reported endorsement with none and/or multiple selections allowed.

	Low Risk	High Risk
Total N	4,142	843
Age (SD)	41.72 (8.33)	41.21 (7.78)
Male	19%	5%
Mean BMI (SD)	29.62 (7.6)	30.91 (9.51)
Mean NIAS Appetite Factor (SD)*	-0.12 (0.85)	0.6 (1.38)
Mean NIAS Fear Factor (SD)*	-0.08 (0.89)	0.39 (1.36)
Mean NIAS Picky Factor (SD)*	-0.1 (0.89)	0.5 (1.31)
Median EAT food time thought (MAD)*	1 (1.48)	2 (1.48)
Median EAT food self control (MAD)*	2 (1.48)	3 (1.48)
Median SENS smell stronger (MAD)*	3 (1.48)	5 (0)
Median EAT small pieces (MAD)*	1 (1.48)	1 (1.48)
Median EAT diet food (MAD)*	1 (1.48)	2 (1.48)
Median EAT guilty (MAD)*	1 (1.48)	2 (1.48)
Median INFLEX eating anxiety (MAD)*	1 (1.48)	3 (2.97)
Median INFLEX specific food present (MAD)*	1 (1.48)	1 (1.48)
Mean GIH routine constipation (SD)*	0.16 (0.37)	0.49 (0.5)
Mean MISC total allergies (SD)*	0.26 (0.7)	0.7 (1.26)

Table S3. Parent demographic summary and ARFID model variables stratified by ARFID risk group. An asterisk * indicates the variable was included in the model. For the individual items included in the model, the raw variables are being reported (unimputed and not corrected for age/sex). Variables with the mean reported have the standard deviation in parenthesis. Variables with the median reported have the median absolute deviation in parenthesis. Nine probands did not have data for cognitive impairment.

Subscale	N	Mean	SD
**DCDQ**			
control during movement	4,064	15.58	5.87
fine motor handwriting	4,064	10.48	4.51
general coordination	4,064	13.96	4.25
DCDQ final	3,965	52.99	12.30
**RBS-R**			
compulsive behavior	4,836	5.45	4.30
restricted behavior	4,838	4.25	2.90
ritualistic behavior	4,837	5.95	4.10
sameness behavior	4,838	9.33	6.34
self injurious	4,831	3.48	3.86
stereotyped behavior	4,831	5.65	3.52
RBS-R final	4,833	34.12	19.54
**SCQ**			
communication	4,452	6.63	2.30
reciprocal interaction	4,385	8.16	3.69
stereotyped behavior	4,456	6.37	2.00
SCQ final	4,615	22.44	6.91
**VABS**			
adaptive behavior composite	1,837	89.84	16.00
communication	1,837	88.93	20.66
community	1,837	15.14	4.09
coping	1,837	14.63	3.61
daily living skills	1,837	87.05	18.15
domestic	1,837	14.67	3.56
expressive	1,837	11.89	4.00
externalizing	1,837	18.49	2.45
internalising	1,837	19.70	1.97
interpersonal	1,837	14.48	3.63
personal	1,837	14.75	3.63
play and leisure	1,837	15.77	3.89
socialization	1,837	90.38	19.63
written	1,837	14.23	4.13
motor	717	79.12	13.33
fine motor	717	14.13	3.32
gross motor	717	10.22	2.68

Table S4. Sample size, mean, and standard deviation of various assessments which were used in association with the NIAS factors and ARFID score
